# People with Multiple Tattoos and/or Piercings Are Not at Increased Risk for HBV or HCV in The Netherlands

**DOI:** 10.1371/journal.pone.0024736

**Published:** 2011-09-14

**Authors:** Anouk T. Urbanus, Anneke van den Hoek, Albert Boonstra, Robin van Houdt, Lotte J. de Bruijn, Titia Heijman, Roel A. Coutinho, Maria Prins

**Affiliations:** 1 Cluster Infectious Diseases, Department of Research, Public Health Service, Amsterdam, The Netherlands; 2 Center for Infection and Immunology Amsterdam (CINIMA), Academic Medical Centre, University of Amsterdam, Amsterdam, The Netherlands; 3 Centre for Infectious Disease Control, National Institute for Public Health and the Environment (RIVM), Bilthoven, The Netherlands; Institut Pasteur, France

## Abstract

**Background:**

Although published results are inconsistent, it has been suggested that tattooing and piercing are risk factors for HBV and HCV infections. To examine whether tattooing and piercing do indeed increase the risk of infection, we conducted a study among people with multiple tattoos and/or piercings in the Netherlands who acquired their tattoos and piercings in the Netherlands and/or abroad.

**Methods:**

Tattoo artists, piercers, and people with multiple tattoos and/or piercings were recruited at tattoo conventions, shops (N = 182), and a biannual survey at our STI-outpatient clinic (N = 252) in Amsterdam. Participants were interviewed and tested for anti-HBc and anti-HCV. Determinants of HBV and HCV infections were analysed using logistic regression analysis.

**Results:**

The median number of tattoos and piercings was 5 (IQR 2–10) and 2 (IQR 2–4), respectively. Almost 40% acquired their tattoo of piercing abroad. In total, 18/434 (4.2%, 95%CI: 2.64%–6.46%) participants were anti-HBc positive and 1 was anti-HCV positive (0.2%, 95%CI: 0.01%–1.29%). Being anti-HBc positive was independently associated with older age (OR 1.68, 95%CI: 1.03–2.75 per 10 years older) and being born in an HBV-endemic country (OR 7.39, 95%CI: 2.77–19.7). Tattoo- and/or piercing-related variables, like having a tattoo or piercing in an HBV endemic country, surface percentage tattooed, number of tattoos and piercings etc., were not associated with either HBV or HCV.

**Conclusions:**

We found no evidence for an increased HBV/HCV seroprevalence among persons with multiple tattoos and/or piercings, which might be due to the introduction of hygiene guidelines for tattoo and piercing shops in combination with the low observed prevalence of HBV/HCV in the general population. Tattoos and/or piercings, therefore, should not be considered risk factors for HBV/HCV in the Dutch population. These findings imply the importance of implementation of hygiene guidelines in other countries.

## Introduction

Hepatitis B virus (HBV) and hepatitis C virus (HCV) can both become chronic infections, causing liver cirrhosis, hepatocellular carcinoma, and eventually death. HBV is transmitted by sexual contact and by blood contact, whereas HCV is primarily a blood-borne infection [1], [2].

Tattooing and piercing have been performed for thousands of years and have been notably popular in recent years. In the Netherlands, an estimated 6% of the general population have at least 1 tattoo and approximately 3% have 1 or more body piercings, excluding regular ear piercings [3].

It has been suggested that people with multiple tattoos and/or piercings are at increased risk for HBV and HCV. However, study results are inconsistent [4]. The association between HBV and tattoos was chiefly found in the 1980s and early 1990s [5]–[7], in more recent literature, rarely is an association found [8], [9]. The association between HCV and tattoos has been inconsistent throughout time [4], [10]–[13], but when there have been associations, they have been found primarily among HCV high-risk populations [4]. Acari et al. reported in their review that none of the studies found an association between body piercing and HCV infection [4].

Tattoo artists and piercers are at possibly even higher risk than their clients for HBV and HCV. They usually have multiple tattoos and piercings themselves, and also because of the lack of sterilization procedures in the past in combination with needle-stick accidents. During an investigation of an HBV outbreak in Amsterdam in 1982, which was traced back to a HBV-infected tattoo artist, many other practices surfaced that likely put clients at risk for infection, such as reusing needles and insufficient or lack of sterilization procedures. As a result of this incident, the Dutch Tattoo Association was set up and developed, in cooperation with the Pubic Health Service of Amsterdam (PHSA), the first set of guidelines for tattoo shops in Amsterdam. More elaborate guidelines were established in 1987 [14]. In 1990, these guidelines were expanded to include piercing and permanent make-up studios.

To the best of our knowledge, no study on HBV and HCV has been performed exclusively among people with multiple tattoos and piercings including tattoo artists. We examined the HBV and HCV prevalence in this population and its relationship with tattoo and piercings practices in a city where guidelines for hygienic practices were introduced two decades ago.

## Methods

### Ethics statement

The medical ethics committee of the Academic Medical Centre (MEC AMC) approved the current study.

### Study population and study procedures

Dutch- or English-speaking tattoo artists and/or piercers living in the Netherlands and people with multiple tattoos and/or piercings (not those with only regular ear piercings) were approached to participate in this study. Participants were recruited at three sites: tattoo conventions in June 2007 and June 2008 in Amsterdam (N = 130); at tattoo and/or piercing shops in Amsterdam (2008–2010) (N = 64); and during two waves of a biannual HIV- and HCV-survey at the sexually transmitted infection (STI) outpatient clinic of Amsterdam in November 2008 and April 2009 (N = 361) [15]. Since recent data have shown that HCV is emerging as an STI among HIV-infected men who have sex with men (MSM) and prevalent in this group [15], all MSM (N = 83) were excluded in this analysis. In addition, to avoid possible confounding, all participants with a history of being an injecting drug user (IDU)(N = 3) were excluded. Participants lacking an HBV core antigen (anti-HBc) or anti-HCV test result (N = 35) were also excluded, leaving 434 participants as our study population.

#### Recruitment at tattoo sites

After we obtained written informed consent, the participants (≥18 years) were interviewed about sociodemographics, risk factors for blood-borne diseases, and about tattoo- and/or piercing-related characteristics; blood was collected for HBV/HCV testing. Subjects were included at the site of the convention by convenience sampling.

Tattoo and/or piercing shops were first approached about the study by a letter, followed by a visit by a PHSA study nurse. During this visit, both clients and tattoo artists and piercers were invited to participate. All participants could choose to receive their HBV and HCV results. In case of positive test results, participants were notified and referred to their general practitioner for further diagnostics and referral to a hospital. Participants received 10 euros as an incentive and anti-HBc negatives received a HBV vaccination series free of cost.

#### Recruitment during biannual survey at the STI outpatient clinic

After written informed consent was obtained, the participants (≥17 years) were interviewed about sociodemographics and risk factors for blood borne diseases and STI and blood samples were collected. When a subject had two or more tattoos and/or piercings, additional information on tattoos and piercings characteristics was gathered, as was done at the tattoo sites. During this survey, however, testing for HCV and HIV was performed anonymously and therefore participants were not notified of the results. STI and anti-HBc status were tested as part of the routine STI clinic consult and these test results were given to participants [16]. Anti-HBc data were merged with the survey data.

### Laboratory testing

Participants were tested for the presence of anti-Hbc and anti-HCV by means of a third-generation commercial microparticle EIA system (AxSym Core, Abbott, Germany and AxSym HCV version 3.0; Abbott, respectively). Participants whose tests confirmed anti-HBc were tested for HBsAg to determine whether they were a chronic carrier. The HBV DNA of HBsAg-positive samples was isolated, amplified, and sequenced, using an in-house PCR as described by van Houdt et al. [17]. Anti-HCV positive samples were confirmed by Immunoblot (Chiron RIBA HCV 3.0 SIA; Ortho-Clinical Diagnostics) and tested for HCV RNA with an in-house PCR, as described by van de Laar et al. [18].

The nucleotide sequence data have been deposited in the GenBank sequence database under accession numbers JN547478–JN547481.

### Variables

Tattoo- and piercing-related variables included the location and number of tattoos and/or piercings, the percentage of the body covered with tattoos, age at first tattoo or piercing, having a tattoo and/or piercing from abroad, as well as information about other established risk factors for acquiring HBV and HCV, such as blood transfusion before 1992, IDU, and sexual anamnesis. For those working as a tattoo or piercing artist, data on the working methods of the tattoo artist and/or piercer were obtained (e.g. using sterile packed needles, working outside the Netherlands, first calendar year of working as an artist, and ever having a needle-stick accident during tattoo or piercing practices). All countries with a moderate to high risk of infection (>2%) in the general population were considered to be HBV or HCV endemic [19], [20].

### Statistical analysis

First, by means of the chi-squared test for categorical variables and the Mann–Whitney test for continuous variables, characteristics of participants who tested positive or negative for anti-HBc and/or anti-HCV were compared. Categories of the variables number of tattoos, % body surface tattooed and number of piercings were defined by cut-off points at the 25^th^, 50^th^ and 75^th^ percentiles for the total group. Confidenc*e* intervals (CI) around prevalence were calculated via the Wilson method, using the binom statistical package in R [21]. Odds ratios (OR) and 95% confidence interval in a 2×2 table with one zero cell count were calculated via penalized logistic regression using the logistf package in R [22]. Otherwise, logistic regression in SPSS 17.0 was used. Multivariate logistic regression models were built using backward stepwise techniques considering variables with a univariate *p*-value≤0.25 as potential independent risk factors. A *p*-value of less than 0.05 was considered statistically significant. Interaction terms were checked in the final model but were not significant.

## Results

### Study population

In total, 434 subjects with multiple tattoos and/or piercings participated in this study. Sixty-three were included during the tattoo convention 2007, 59 during the tattoo convention 2008, 60 at tattoo and piercing shops and 252 at the STI clinic in Amsterdam. Of all the subjects, 187 (43.1%) were male and the median age was 28 years (IQR 23–37 years) (Table 1). More than half of the population reported that they had not been vaccinated against HBV (57.9%). The median number of tattoos and piercings were 5 (IQR 2–10) and 2 (IQR 2–4) respectively. The median body surface reported by the participants as tattooed was 18% (IQR 9%–27%). Participants included at tattoo venues were significantly more often men, older, with higher education, and had a larger number of tattoos and piercings compared to the participants who were included at the STI clinic.

**Table 1:** Characteristics of 434 participants with multiple tattoos and piercings, stratified by anti-Hbc status#.

	Total	Anti-Hbc negative	Anti-Hbc positive	Univariate analysis	*P*-value*	Multivariate analysis	*P*-value*
	N = 434 (%)	N = 416 (%)	N = 18 (%)	OR (95% CI)			
***Social demographics and relevant background information***							
**Median age (IQR) (per 10 years older)**	28 (23–37)	28 (22–36)	32.5 (25–44)	1.65* (1.05–2.59)	0.03	1.68 (1.03–2.75)	0.03
**Gender**							
Female	247 (56.9%)	241 (57.9%)	6 (33.3%)	1	0.05		
Male	187 (43.1%)	175 (42.1%)	12 (66.7%)	2.75 (1.01–7.48)			
**Education**							
Low	123 (28.5%)	116 (28.0%)	7 (41.2%)	1	0.25		
Middle	181 (41.9%)	173 (41.6%)	8 (47.1%)	3.80 (0.77–18.7)			
High	128 (29.6%)	126 (30.3%)	2 (11.8%)	2.91 (0.61–13.9)			
Missing	2	1	1				
**Residence**							
Amsterdam	317 (74.2%)	305 (74.6%)	12 (66.7%)	1	0.46		
Other	110 (25.8%)	104 (25.4%)	6 (33.3%)	1.46 (0.54–4.01)			
Missing	7	7					
**Born in HBV endemic country**							
No	364 (83.9%)	356 (85.6%)	8 (44.4%)	1	<0.001	1	<0.001
Yes	70 (16.1%)	60 (14.4%)	10 (55.6%)	7.42 (2.81–19.6)		7.39 (2.77–19.7)	
**Recruitment site**							
STI outpatient clinic	252 (58.1%)	241 (57.9%)	11 (61.1%)	1	0.79		
Tattoo venue	182 (41.9%)	175 (42.1%)	7 (38.9%)	0.88 (0.33–2.31)			
**HBV vaccination (self-reported)**							
No	249 (57.9%)	237 (57.4%)	12 (70.6%)	1	0.21		
Yes	136 (31.6%)	134 (32.4%)	2 (11.8%)	0.30 (0.07–1.34)			
Don't know	45 (10.5%)	42 (10.2%)	3 (17.6%)	1.41 (0.38–5.21)			
Missing	4	3	1				
**HCV status**							
Negative	433 (99.8%)	415 (99.8%)	18 (100%)	1	0.39		
Positive	1 (0.2%)	1 (0.2%)	0	7.49 (0.08–708.7)			
**Snorting drugs**							
No	243 (63.1%)	229 (62.2%)	14 (82.4%)	1	0.11		
Yes	142 (36.9%)	139 (37.8%)	3 (17.6%)	0.35 (0.10–1.25)			
Missing	49	48	1				
***Tattoo and piercing characteristics***							
**Being a tattoo/piercing artist**							
No	345 (80.6%)	332 (80.8%)	13 (76.5%)	1	0.66		
Yes	83 (19.4%)	79 (19.2%)	4 (23.5%)	1.29 (0.41–4.07)			
Missing	6	5	1				
**Tattoo and piercing**							
Piercing	59 (13.6%)	58 (13.9%)	1 (5.6%)	1	0.19		
Tattoo	114 (26.3%)	106 (25.5%)	8 (44.4%)	4.37 (0.53–35.9)			
Tattoo and piercing	261 (60.1%)	252 (60.6%)	9 (50.0%)	2.07 (0.26–16.7)			
**Number of tattoos**							
No tattoo	59 (13.6%)	58 (13.9%)	1 (5.6%)	1	0.77		
1–2	106 (22.4%)	101 (24.3%)	5 (27.8%)	2.87 (0.32–25.2)			
3–4	91 (21.0%)	88 (21.2%)	3 (16.7%)	1.98 (0.20–19.5)			
5–10	96 (22.1%)	92 (22.1%)	4 (22.2%)	2.52 (0.28–23.1)			
>11	82 (18.9%)	77 (18.5%)	5 (27.8%)	3.77 (0.43–33.1)			
**% body surface tattooed**							
No tattoo	59 (13.6%)	58 (13.9%)	1 (5.6%)	1	0.61		
1%–9%	116 (26.7%)	113 (27.2%)	3 (16.7%)	1.54 (0.16–15.1)			
10%–18%	117 (27.0%)	110 (26.4%)	7 (38.9%)	3.69 (0.44–30.7)			
19%–31.5%	68 (15.7%)	65 (15.6%)	3 (16.7%)	2.67 (0.27–26.5)			
>31.5%	74 (17.0%)	70 (16.8%)	4 (22.2%)	3.31 (0.36–30.5)			
**Tattoo and/or piercing in HBV endemic country**							
Tattoo/piercing in low endemic country	322 (74.2%)	310 (74.5%)	12 (66.7%)	1	0.46		
Tattoo/piercing in endemic country	112 (25.8%)	106 (25.5%)	6 (33.3%)	1.46 (0.54–3.99)			
**Number of piercings**							
No piercing	114 (26.2%)	106 (25.5%)	8 (44.4%)	1	0.36		
1	65 (15.0%)	64 (15.6%)	1(5.6%)	0.21 (0.03–1.69)			
2	104 (24.0%)	99 (23.8%)	5 (27.8%)	0.68 (0.21–2.14)			
3–4	72 (16.6%)	71 (17.1%)	1(5.6%)	0.19 (0.02–1.53)			
≥5	79 (18.2%)	76 (18.3%)	3 (16.6%)	0.52 (0.13–2.04)			

Univariate and multivariate analysis logistic regression analysis.

# Men who have sex with men (MSM) and injecting drug users (IDU) are excluded;

*overall *p*- value.

### Hepatitis B

In total 18/434 (4.2%, 95% CI; 2.64–6.46) participants were found to be anti-HBc positive and were considered as having had a HBV infection (Table 1). The country of birth of the anti-HBc positives were; South-Africa, Aruba, Surinam (n = 7), Indonesia, Germany, and the Netherlands (n = 7 of whom one had Ghanaian parents and one had Vietnamese parents). Three of the 18 anti-HBc-positive participants were chronic carriers (HBsAg positive), which is 0.7% (3/434, 95% CI: 0.24–2.01) of the total study population. In univariate analysis, older age, male sex, and born in an HBV-endemic country were associated with anti-HBc seropositivity (Table 1). None of the tattoo-related variables, including the number of tattoos and piercings, percentage of the body surface tattooed, having a tattoo in an HBV-endemic country or being a tattoo artist, were significantly associated with HBV. In multivariate analysis, older age and being born in an HBV-endemic country were independently associated with anti-HBc seropositivity (OR 1.68; 95% CI: 1.03–2.75 per 10 years older and OR 7.39; 95% CI: 2.77–19.7 respectively). Tattoo artists appeared to be more likely to have been vaccinated against HBV (self-reported) than those who were not tattoo artists (OR 2.52; 95% CI: 1.49–4.24).

An additional analysis, conducted among tattoo and piercing artists only, confirmed that none of the tattoo and/or piercing variables were significantly associated with HBV. Restricting our analysis to unvaccinated participants, again no tattoo- and/or piercing-related variables were associated with HBV.

After sequencing the viral HBV DNA of the three chronic carriers, two proved to be infected with genotype A. One of the two was German, the other was Surinamese. Neither reported a high number of lifetime sexual partners, but they did have multiple tattoos (both N = 15). The other chronic carrier was infected with genotype B and of Indonesian ethnicity, with only a few tattoos (N = 4) and a small number of lifetime sexual partners, and might have been infected in the country of birth.

### Hepatitis C

Only one participant was HCV infected (0.2%, 95% CI: 0.01–1.29) with genotype 1a. This participant was a tattoo artist who received a tattoo more than 100 times, and reported several other potential risk factors for HCV, including needle-stick accidents. In phylogenetic analysis, this strain did not cluster with MSM-, IDU-specific or endemic clusters (data not shown).

## Discussion

In this study, we did not find any association between tattoo or piercing characteristics and HBV or HCV infection. The HCV prevalence in this study was low (0.2%, 95% CI: 0.01–1.29) and comparable to the prevalence of the general Dutch population (0.1–0.4%) [23]. The HBsAg prevalence and anti-HBV prevalence in the Netherlands are estimated to be 0.3–0.5% and 2.1% (95% CI: 1.6–2.7), respectively [24], [25]. In our study, the HBsAg and anti-HBc prevalences were 0.7% and 4.2%, respectively, which is in line with the estimates of the Amsterdam population (0.4%, 95% CI: 0.11–0.72 and 9.9%, 95% CI: 1.0–8.0 respectively) which are somewhat higher due to the higher migration rates in Amsterdam [26]. Comparing participants having a tattoo and participants not having a tattoo in a population based survey in Amsterdam [26] we found no differences in HBV and HCV prevalence between the two groups (data not shown). The same accounts for the larger population of participants in the biannual survey at the STI-outpatient clinic, which is one of the recruitment sites (data not shown). Based on these findings together with the fact that we did not found a dose-response relationship between tattoo characteristics such as the number of tattoos or piercings and the percentage body surface tattooed and HBV or HCV infection, we conclude that people with multiple tattoos and/or piercings are not a risk group for HBV or HCV in the Netherlands.

HCV is mainly transmitted by blood-blood contact and main risk factors in high income countries are IDU and receiving blood or blood products before 1992 [1], [27], [28]. The transmission route for HBV are blood-blood contact and sexual contact. The main risk factors in low endemic counties are unsafe sex, and injecting drug use, whereas in intermediate and high endemic countries most infections are acquired perinatally or during early childhood [2]. Since most anti-HBc positive participants (67%, adult first or second generation) originated from intermediate or high endemic countries, and born in an HBV endemic country was significantly associated with HBV, it is likely that these participants were infected in the country of birth, and not by sexual transmission in the Netherlands, which was more expected in attendees of a STI outpatient clinic.

In Amsterdam, for two decades already, guidelines for hygienic practices have been implemented in tattoo and piercing shops. Based on the Amsterdam guidelines, the Dutch government implemented a nationwide law in June 2007 that states that all tattoo and piercing shops in the Netherlands are obliged to have a licence, which has to be obtained yearly and has to be permitted by a local Public Health Service [14], [29]. Since most participants included in this study were living in the region of Amsterdam (74.2%), it is possible that the transmission of HBV and HCV originating in tattoo and piercing shops in Amsterdam has been curtailed by the early implementation of hygienic guidelines. An alternative explanation is that the probability of introduction of HBV and HCV in the tattoo population might be very small due to the low HBV/HCV prevalence in the general Dutch population.

In the Netherlands, having a tattoo or piercing is currently defined as a risk factor for HBV/HCV and screening for the presence of both viruses in this group is recommended [30]. However, our findings suggest that for persons having a tattoo or piercing in a country where guidelines for hygiene practices have been available for a considerable time period, this screening should not routinely be advised.

Arcari et al. [4] concluded in their review that in most studies IDU is a major confounding factor. Most often, when there is an association found, there is no information available about the tattoo venue and other tattoo characteristics (e.g. number) in these studies [4] and therefore residual confounding could play a role. To our best knowledge, our study is the only study worldwide exclusively performed in people with multiple tattoos and piercings who were not IDU or MSM. The fact that we did not find any association suggests that residual confounding might indeed have played a role in previous studies. However, our findings do not apply to populations with a high HBV/HCV prevalence or to countries without hygiene guidelines.

In conclusion, in low HBV/HCV-endemic countries where strict hygiene guidelines for tattoo and piercing practices have been implemented, like in the Netherlands, tattoo and piercing practices are not associated with HBV/HCV infection and people with tattoos and/or piercings should not be advised to be screened for HBV and HCV in order to trace undiagnosed infections. We recommend low and high endemic countries to implement hygienic guidelines for tattoo and piercing shops, including permanent make-up salons, to decrease the potential risk of HBV and HCV transmission. More studies are needed to generalize our findings, and should be conducted exclusively among people with multiple tattoos and piercings in both low and high endemic countries that exclude IDU and MSM but include information on tattoo and piercing practices, as well as hygiene guidelines.
